# Antitumoral Drug-Loaded Biocompatible Polymeric Nanoparticles Obtained by Non-Aqueous Emulsion Polymerization

**DOI:** 10.3390/polym12051018

**Published:** 2020-04-30

**Authors:** Oana Maria Daraba, Anca Niculina Cadinoiu, Delia Mihaela Rata, Leonard Ionut Atanase, Gabriela Vochita

**Affiliations:** 1 Department of Biomaterials, Faculty of Medical Dentistry, “Apollonia” University of Iasi, Pacurari Street, No. 11, Iasi 700511, Romania; maria.mary2019@yahoo.com (O.M.D.); jancaniculina@yahoo.com (A.N.C.); iureadeliamihaela@yahoo.com (D.M.R.); 2 Department of Experimental and Applied Biology, NIRDBS—Institute of Biological Research Iasi, Lascar Catargi 47, Iasi 700107, Romania; gabriela.vochita@icbiasi.ro

**Keywords:** poly(ε-caprolactone), poly(N-vinylpyrrolidone), silicon oil (PDMS), nanoparticles, Cisplatin, non-aqueous emulsion polymerization, apoptosis

## Abstract

Non-aqueous dispersions (NAD) with two types of polymeric nanoparticles (NPs), such as hydrophobic poly(*ε*-caprolactone) (PCL) and hydrophilic cross-linked poly(vinylpyrrolidone) (PNVP), were synthesized in the present study starting from monomer-in-silicone oil (PDMS) polymerizable non-aqueous emulsions stabilized with the same tailor-made PDMS-based block copolymer. These NPs were loaded with CCisplatin, an antitumoral model drug, directly from the emulsion polymerization step, and it was observed that the presence of the drug leads only to a slight increase of the NPs size, from 120 to 150 nm. The drug release kinetics was evaluated at 37 °C in phosphate buffer at pH = 7.4 and it appeared that the drug release rate from the hydrophilic cross-linked PNVP-based NPs is higher than that from the hydrophobic PCL-based NPs. Moreover, haemolysis tests revealed the fact that these two types of NPs have a good compatibility with the blood. Furthermore, for both the free and drug-loaded NPs, the *in vitro* cytotoxicity and apoptosis was studied on two types of cancer cell lines, such as MCF-7 (breast cancer cell line) and A-375 (skin cancer cell line). Both types of NPs had no cytotoxic effect but, at a concentration of 500 μg/mL, presented an apoptotic effect similar to that of the free drug.

## 1. Introduction

Controlled drug delivery (CDD) has become one of the important research areas in polymeric biomaterials during the past few years [1,2,3,4,5]. Such delivery systems offer numerous advantages compared to conventional dosage forms including improved efficiency, reduced toxicity, and increased patient compliance [3,4]. In this respect, a wide range of drug-loaded nanoparticles, with diameters between 10 and 1000 nm, have been used as CDD systems and their advantages over the microparticles are well highlighted [6,7]. The nanoparticles (NPs), that can be either nanospheres or nanocapsules, are based on biocompatible polymers and their advantages are: high drug encapsulation efficiency; improved drug bioavailability; solubility and retention time; enhanced chemical and biological stability; controlled drug release rate; and wide variety of administration routes. The drug release rate from these biodegradable polymeric NPs is controlled by the biodegradation kinetics of the polymers, the physicochemical properties of the polymers and drugs, the thermodynamic compatibility between the polymers and drugs.

For the preparation of the drug-loaded NPs, two methods are described in the literature: an emulsion polymerization and directly from preformed polymers [6,7,8,9]. For this last method, various polymeric materials are commonly used such as poly (lactic acid) (PLA), poly(glycolic acid) (PGA), poly(lactic-co-glycolic acid) (PLGA), poly(*ε*-caprolactone) (PCL), poly(N-vinyl-pyrrolidone) (PNVP), or natural polymers.

It is worth noting that the synthesis of NPs by emulsion polymerization has an advantage with respect to the methods using the preformed polymers in solution, such as nanoprecipitation or emulsification-diffusion, because it is a one-pot process that allows high-solid-content latex without any further concentration step and a better control of the NPs size. The emulsion polymerization can be classified in two categories, aqueous and non-aqueous emulsions, based on the use of either an organic or an aqueous continuous phase. Drug-loaded NPs of cyanoacrylates, methacrylates, malonates, and vinyl esters have been obtained by aqueous miniemulsion polymerization [10,11,12,13,14]. Even if this polymerization process has a number of advantages, it cannot be used if water-sensitive mechanisms, such as anionic ring-opening polymerization (ROP) of *ε*-caprolactone (CL), are employed. In this case, a non-aqueous emulsion polymerization is mandatory. Recently, this concept has received increasing interest, as shown by the groups of Klapper [15] and Landfester [16,17], for a wide range of water-sensitive systems. For instance, Ruppert et al. [18] have achieved the anionic polymerization of CL in a hydrocarbon solvent in the presence of DMSO as cosolvent but such a process is not suitable for biomedical applications. Using a similar hydrocarbon solvent system, Crespy and Landfester [19] have succeeded to prepare poly(N-vinyl-pyrrolidone) (PNVP)/silver nanoparticles hybrid latex by free radical polymerization in inverse miniemulsion at high temperatures. At this point, it is also of interest to notice the study of Hariri et al. [20] on the non-aqueous emulsion polymerization of VP in a functionalized silicon oil (PDMS) phase by generating in situ a graft copolymer as stabilizer. However, for such a typical biocompatible system, the graft copolymer was not suitable to provide the emulsion stability and the particle size control. 

The general aim of this study was to develop new drug delivery systems of controlled particle size (smaller than 200 nm) based on PCL and cross-linked PNVP NPs prepared by a biocompatible non-aqueous emulsion polymerization. In this type of emulsion, usually designated as oil-in-oil (O/O) emulsion, the monomer droplets are dispersed in a non-miscible oil and the stabilization can be achieved by using tailor-made copolymers. It has been shown that copolymers can provide longer stability for O/O emulsions with respect to the conventional surfactants because they have a much slower desorption kinetics at the interface of the droplets [21,22,23,24]. Moreover, it is well known that PNVP, which is FDA approved, is a hydrophilic polymer and has a good efficiency to control the release rate of poorly water-soluble drugs [25]. In order to assure the integrity of these PNVP-based NPs in aqueous medium, a cross-linking agent was used at different concentrations [26]. On the other hand, PCL, also approved by the FDA, is characterized by a high hydrophobicity, biocompatibility, biodegradability, non-toxicity, and it is permeable to low-molecular-weight drugs [27,28]. Furthermore, these biocompatible NPs were loaded with Cisplatin (Cis), a model antitumoral drug, which is commonly used for the treatment of several cancers, such as skin, breast, and lung [29,30].

The size of both the non-aqueous emulsion and non-aqueous dispersion was analyzed by DLS. Having in mind that the PNVP-based NPs are hydrophilic, it was of interest to study, the swelling degree of the NPs at 37°C and at a pH value of 7.4. For both types of NPs, in vitro drug release rate was further investigated at 37°C in phosphate buffer at pH = 7.4. Moreover, the influence of the obtained NPs on the blood components was studied by the determination of the hemocompatibility. Furthermore, the in vitro cytotoxicity and apoptosis assays were studied on two types of cancer cell lines, such as MCF-7 (breast cancer cell line) and A-375 (skin cancer cell line).

## 2. Materials and Methods

### 2.1. Materials

N-vinyl-2-pyrrolidone (VP) (Sigma-Aldrich; Steinheim, Germany, 99%) was purified by passing through a column of aluminum oxide. 2,2′-azobis(isobutyronitrile) was recrystallized from methanol prior to use. *ε*-caprolactone (CL) was purchased from Sigma-Aldrich (Steinheim, Germany), dried over CaH_2_ and distilled at reduced pressure. Phosphotungstic acid was purchased from Sigma-Aldrich (Steinheim, Germany) and dried at 150 °C for 2 h before use. Hydroxyl terminated poly(dimethylsiloxane) (PDMS-OH, *M*_n_ = 4900 g/mol, X-22-170-DX, ShinEtsu, Tokyo, Japan) was dried over molecular sieves (3Å). Triethyl aluminum (TEA, 0.9 M in hexane, purum, Fluka, Buchs, Switzerland) was used without further purification. Toluene (99.5%, Fluka, Buchs, Switzerland) was distilled over CuCl/NaOH and over sodium, and dried over molecular sieves (3Å). The following chemical products were used without purification: 4,4′-azobis(4-cyanopentanoic acid) (ACPA) (Sigma-Aldrich, Steinheim, Germany), silicone oil 47 V50 (Rhodorsil) (VWR), N,N’-methylenebis(acrylamide) (Sigma-Aldrich, Steinheim, Germany), Cis-diamminedichloroplatinum(II), Pt 64.5% (Cisplatin) (Alfa Aesar, Karlsruhe, Germany). Human breast carcinoma MCF7 (ATCC^®^HTB-22D™) and A375 human malignant melanoma cells (ATCC CRL-1619) were purchased from ATCC^®^ (Manassas, VA, USA). The necessary supplies for in vitro cytotoxicity assay (Dulbecco’s modified Eagle medium-DMEM, streptomycin and penicillin were purchased from Biochrom AG, Berlin, Germany. Fetal bovine serum (FBS) was acquired from Sigma-Aldrich, Steinheim, Germany. 

### 2.2. Synthesis of PCL-b-PDMS Diblock Copolymer

The polymerization was carried out in a previously dried Schlenk tube equipped with a magnetic stirring bar under nitrogen. The tube was degassed three times by repeated vacuum/nitrogen cycles. PCL-b-PDMS diblock copolymer was synthesized as following: into a Schlenk tube were added 10 g (2.04 mmol) of hydroxyl terminated PDMS, 0.23 mL (0.204 mmol) of triethyl aluminum, and 200 mL of dry toluene. The reaction mixture was stirred at 55 °C, under nitrogen for 2 h. Then 8.66 mL (81.6 mmol) of CL were added to the mixture, and the reaction was allowed to proceed for 2 h at 55 °C. Finally, 4 mL of ethyl alcohol were added for stopping the reaction and the copolymer was precipitated into heptane, filtered and dried under vacuum. Copolymer PCL_5200_-*b*-PDMS_4900_ (the subscript represent the *M*_n_ obtained by ^1^H NMR): *M*_n_(SEC) = 1,0100 g/mol; PDI = 1.17; conversion = 100% for CL(SEC).

### 2.3. Non-Aqueous Emulsion Preparation and Characterization

The O/O emulsions, with the VP or CL monomer as dispersed phase and the silicon oil as continuous one, were prepared by mechanical stirring (Ultra-turrax T25 IKA) at 10,000 rpm during 5 min at a fixed volume ratio of 10/90 in the presence of 1 and 3 wt % of the tailor-made PCL-PDMS copolymer which was solubilized previously in the PDMS phase. The static stability of the obtained emulsions was determined by following the creaming and/or sedimentation as a function of time. A characteristic stability indication is the *t*_25_ value, which is the time where 25% of the dispersed or the continuous phase volume has separated. The average sizes of the dispersed VP or CL droplets were determined using a dynamic light scattering particle size analyzer (Nanotrac, Microtrac, Osaka, Japan) after dilution with the silicon oil. The values provided in the Table 1 are the average of five consecutive measurements. 

### 2.4. Non-Aqueous Emulsion Polymerization and Characterization

The polymerization of the dispersed VP or CL phase was carried out in a thermostated 100 mL glass reactor equipped with an anchor impeller, a nitrogen inlet and a condenser. For the polymerization of the VP, both the initiator (ACPA) and the cross-linking agent (MBAA) were solubilized in the dispersed monomer phase before the preparation of the non-aqueous emulsion, as previously described. Hariri et al. [20] have demonstrated that ACPA is more efficient than lauroyl peroxide as initiator of the VP emulsion polymerization. The reactor was charged at room temperature with 50 mL of the previously obtained non-aqueous emulsions and the polymerization was carried out during 5 h at 60 °C under agitation (150 rpm). The PNVP particles were separated from the NAD by repeated washing with n-hexane, filtration and drying under vacuum.

The polymerization of the CL was realized in the presence of the PTA at 40 °C over 4 h starting from the previously obtained non-aqueous emulsion. The PCL particles were separated by repeated washing with diethyl ether and methanol, filtration, and drying under vacuum. The physicochemical characterization of the PNVP and PCL polymers are given in Supplementary Materials (Figure S1 and Tables S1–S3).

The Cis-loaded particles were obtained using a similar procedure with the difference that the drug was solubilised in the dispersed VP or CL phase before the emulsification step.

For both NAD systems, the average particle size and the particle size distribution were determined using dynamic light scattering (Nanotrac, Microtrac, Osaka, Japan) after dilution with silicon oil. The values provided in the Table 1 are the average of five consecutive measurements. 

### 2.5. Swelling Behavior in Aqueous Solutions

The swelling kinetics of the obtained NPs in aqueous medium at pH 7.4 was carried out by gravimetric method. A specific amount of dry sample (*W*_0_) was suspended in an aqueous medium. The obtained suspension was introduced in an Eppendorf^®^ tube and was maintained under a magnetic stirring at 120 rpm at room temperature. At specific time intervals the suspension was centrifugated, the supernatant was removed and the swollen sample (*W*_1_) was weighed. The traces of water on the tube walls were eliminated by blotting with filter paper. The swelling degree was calculated using Equation (1)

(1) $$Q\left(\%\right)=\frac{W_{1\;}-{\;W}_{0}}{W_{0}}\times 100$$

### 2.6. In Vitro Drug Release

In vitro release kinetics assays in slightly alkaline aqueous medium with pH 7.4 were performed using a vertical Franz diffusion cell apparatus (Copley vertical diffusion cells system) based on two compartments which were separated by a cellulose artificial membranes. The membrane separates the donor compartment, containing either suspension of nanoparticles loaded with Cis (30 mg of nanoparticles dispersed in 0.5 mL buffer solutions) or free Cis (dissolved in 0.5 mL buffer solutions), from the receptor compartment filled with 7 mL collection medium (buffer solutions at pH 7.4 and 37 ± 1 °C). The mean amount of CCis loaded into nanoparticles was 0.01mg Cis/1 mg nanoparticles and an equivalent dose was used for free Cis. The receptor fluid was thoroughly stirred during the entire experiment. After elapsed times, 0.2 mL of the receiving solution was withdrawn and replaced with an equal volume of prethermostated fresh medium. Sink conditions were maintained throughout the experiment and all experiments were performed in triplicate. The concentration of drug in the receiver medium was determined by UV–Vis spectroscopy (Nanodrop One UV-Vis Spectrophotometer, Thermo Fischer Scientific, Walltham, MA, USA). The release efficiency of CCis (Ref%) was calculated using Equation (2)

(2) $$\mathrm{Ref}\%\;=\frac{m_{r}}{m_{l}}\times \;100$$

where, m_r_ is the released amount of Cis (mg) and m_l_ is the amount of loaded Cis (mg).

### 2.7. In Vitro Hemolysis Assay

The hemolytic potential of nanoparticles was evaluated using a spectrophotometric method adapted from Rață et al. [31]. In these assays, blood from healthy human volunteer was collected and treated with nanoparticles, after institutional ethical clearance and appropriate informed consent. 5 mL of anti-coagulated blood was centrifuged at 2000 rpm (RCF = 381× *g*) for 5 min and washed with normal saline solution several times to completely remove plasma and obtain erythrocytes. Then, the purified erythrocytes were re-suspended in normal saline solution to obtain 25 mL of erythrocytes suspension. 2 mL of NPs suspension in normal saline solution at different concentrations were added to 2 mL of erythrocytes suspension (final concentrations were 100, 250, and 500 mg NPs/mL). Positive (100% lysis) and negative (0% lysis) control samples were prepared by adding equal volumes (2 mL) of Triton X-100 and normal saline solution. The samples were incubated at 37 °C for 90 and 180 min. The samples were slightly shaken once every 30 min to re-suspend the erythrocytes and nanoparticles. After the incubation time, the samples were centrifuged at 2000 rpm (RCF = 381× *g*) for 5 min and 100 µL of supernatant was incubated for 30 min at room temperature to allow hemoglobin oxidation. Oxyhemoglobin absorbance in supernatants was measured spectrophotometrically (Nanodrop One UV–Vis Spectrophotometer) at 540 nm. All samples were analyzed in triplicate. The hemolytic percentage was calculated using Equation (3)

(3) $$Hemolysis\;\left(\%\right)=\frac{\left(Absorbance_{sample}-Absorbance_{negative\;control}\right)}{\left(Absorbance_{pozitive\;control}-Absorbance_{negative\;control}\right)}\;\times \;100$$

### 2.8. Cell Culture

Both cell lines, human breast carcinoma MCF7 (ATCC^®^HTB-22D™) and A375 human malignant melanoma cells (ATCC CRL-1619), were cultured in Dulbecco’s modified Eagle medium (DMEM, Biochrom AG, Berlin, Germany), supplemented with 10% fetal bovine serum (FBS, Sigma, Germany), 100 μg/mL streptomycin (Biochrom AG, Berlin, Germany), 100 IU/mL penicillin (Biochrom AG, Berlin, Germany) in a humidified atmosphere and presence of 5% CO_2_, at 37 °C in order to obtain the <90% confluence in the 75 cm^2^ flasks.

### 2.9. Determination of the Cell Viability by the MTT Method

The effect of the obtained drug delivery systems on cancer cell survival was determined by MTT test according to Mosmann [32] and Laville et al. [33]. This test is based on the ability of living cells to convert the water-soluble yellow substrate [3-(4,5-dimethyl-2-thiazolyl)-2,5-diphenyl-2H-teyrazolium bromide] into the purple formazan product. The amount of insoluble formazan is directly proportional to the number of live cells [34]. In brief, the cells were trypsinized according to standard trypsinization procedure using trypsin/EDTA solution, then counted and re-suspended in 96-well microplates (a density of 8 × 10^3^ cells/well), in the same temperature and humidity conditions. For each samples, the total volume media was 300 μL/well. After monolayer formation (24 h), the cells were treated for 24 and 48h with unloaded and drug-loaded NPs in different doses ranging from 100 to 500 μg/mL. Likewise, the cells were treated with Cis in a corresponding dose with the highest one used in the case of NPs (500 μg/mL). After treatment, the samples were processed by MTT assay, the absorbance being measured at 570 nm using the Biochrom EZ Read 400 microplate automatic reader. The cell viability percentage was calculated according to Equation (4)

(4) $$\mathrm{Cell}\;\mathrm{viability}\;\left(\%\right)=\frac{\mathrm{Absorbance}\;\left(\mathrm{test}\right)}{\mathrm{Absorbance}\;\left(\mathrm{control}\right)}\times \;100$$

### 2.10. Apoptosis Assay

The apoptosis was investigated at 48 h after the treatment by Annexin V-FITC/propidium iodide assay [35]. Investigation of apoptosis by Annexin V/propidium iodide resides in the strong affinity of Annexin V for phosphatidylserine residues on the surface of the cell, which normally are hidden within the plasma membrane. Throughout apoptosis process, the phosphatidylserine is translocated from the inner plasma membrane to the outer cell surface. Propidium iodide fluorocrome is capable of binding to DNA allowing differentiation between living and dead cells, and also separation between preapoptotic and apoptotic cells when it is associated with Annexin V [36,37]. The cells were cultivated in 12 well plates (7.5 × 10^4^ cells/well) in 2 mL/well of total culture media. After trypsinization, the cells were washed with cold phosphate-buffered saline, re-suspended in 400 μL binding buffer, then are marked with 5 μL Annexin V-FITC. After incubation (15 min in the dark), 5 µl propidium iodide (1 mg/mL) were added, in accordance with the manufacturer’s instructions (eBioscience kit). Within 15 min, the analysis of apoptosis was performed with a Beckman Coulter Cell Lab QuantaSC flow cytometer, supplied with a 488 nm laser and the fluorescence was collected for FITC on FL1 (525 nm band pass filter) and for propidium iodide on FL3 (670 nm long pass filter). All data were exported as LMD files and analyzed by FCSalyzer software.

### 2.11. Statistical Analysis

All in vitro experiments were performed on the basis of three repetitions and statistically analyzed using the Student’s *t*-test. The values are expressed as mean ± SE of three parallel measurements.

## 3. Results and Discussion

### 3.1. Non-Aqueous Emulsions and Dispersions

A series of polymerizable non-aqueous emulsions were prepared having as dispersed phase two monomers, such as: vinylpyrrolidone (VP) and *ε*-caprolactone (CL), and as continuous phase a biocompatible silicon oil (PDMS). The stabilization of these non-aqueous emulsions was assured by a tailor-made block copolymer at different concentrations. In order to prepare drug delivery systems, a model antitumoral drug (Cis) was solubilized in the dispersed phase which was further polymerized. The stability of these emulsions was determined by following the creaming and/or sedimentation as a function of time. A characteristic stability indication is the *t*_25_ value, which is the time where 25% of the dispersed (VP or CL) or continuous PDMS volume has phase separated. Preliminary tests revealed, as expected, that with a copolymer concentration of 3 wt % (*t*_25_ = 24 h at 25 °C) more stable non-aqueous CL or VP-in-PDMS emulsions are obtained than at a copolymer concentration of 1 wt % (*t*_25_ = 2 h at 25 °C).

Table 1 presents the droplet and particle sizes as well as the polydispersity indexes (PDI) for both the non-aqueous emulsions and dispersions.

From Table 1, it can be observed that the size of both types of NPs, obtained by non-aqueous polymerization of the dispersed VP or CL phase, shows a slight increase compared to the size of the starting non-aqueous emulsion. Moreover, the PDI of all the samples, both non-aqueous emulsions and dispersions, is very low. This is a clear evidence that the initial emulsion remained stable throughout the polymerization and that the copolymer used is an effective stabilizer. It also appears that the solubilization of the Cis in the dispersed phase has as consequence an increase of both the size of droplet and NPs. Nevertheless, these sizes are under 150 nm and therefore these systems can be used as injectable drug delivery systems.

### 3.2. Swelling Behavior

**Figure 1:**
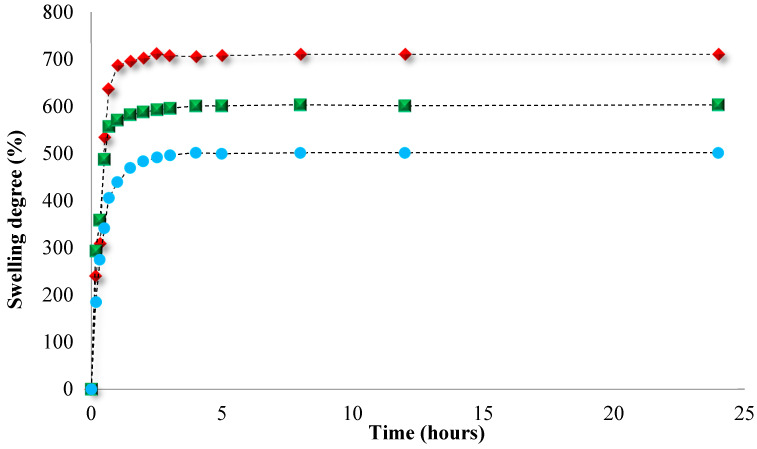
Swelling kinetics curves of the PNVP-based NPs in alkaline conditions (pH = 7.4). Circles—NPs3; Squares—NPs2; Diamonds—NPs1.

Knowing that the PCL-based NPs are hydrophobic in nature, the swelling behavior was investigated only for the hydrophilic cross-linked PNVP-based NPs in slightly alkaline medium (PBS = pH 7.4) and the obtained results are illustrated in Figure 1**.** This medium was chosen as these drug delivery systems are intended to be used in biological fluids. 

Figure 1 shows that the swelling degree of the PNVP-based NPs in slightly alkaline medium varies from 490% to 660% as a function of the concentration of the cross-linking agent. Moreover, it can be observed that the analyzed samples exhibited a rapid swelling, which is caused by the water penetration by diffusion within the NPs structure until their filling, followed by slower swelling of polymeric network until equilibrium (generally after about 3 h).

### 3.3. In Vitro Release of Cis

**Figure 2:**
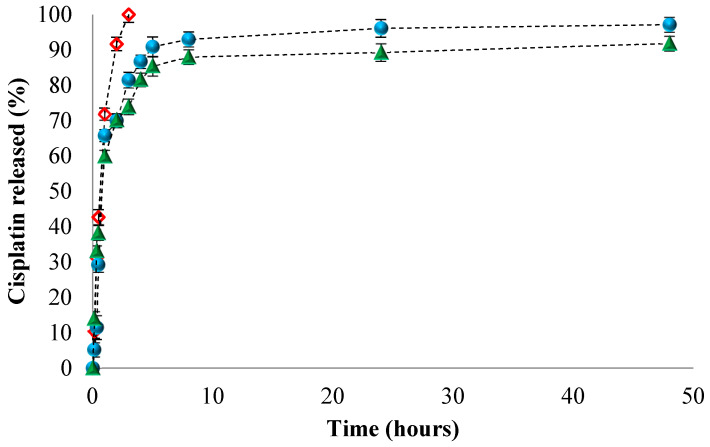
In vitro release kinetics of free (diamonds) and Cis loaded in the PNVP (NPs4) (full circles) and PCL-based (NPs6) (full triangles) NPs in phosphate buffer solution (pH 7.4).

Both PCL and PNVP polymers have been already used for the preparation, by a precipitation method, of drug-loaded NPs. However, as far as one can tell from the literature, the preparation, by a biocompatible emulsion polymerization method, of CDD systems based on PCL of PNVP polymers has not been yet reported. The practical application of the systems obtained in this study might be the treatment of cancer. Therefore, the drug encapsulated in these NPs was Cisplatin (Cis) which is one of the most potent and widely-used anticancer drug for the treatment of a variety of solid tumors: ovarian, testicular, bladder, cervical, head and neck, esophageal, and small-cell lung cancers [28,29]. The biological activity is derived from its interactions with DNA, Cis interfering with the normal transcription and replication processes resulting cell death [38,39]. The release studies of both the free and Cis loaded in the NPs4 (PNVP-based) and NPs6 (PCL-based) were performed in slightly alkaline medium (pH = 7.4) and the release efficiencies are presented in Figure 2**.**

From Figure 2, it appears that 100% of the free Cis crosses the dialysis membrane after approximately 3h. For the Cis loaded in both types of NPs, a faster release was observed in the first 4h which may be due to the phenomenon of drug desorption from the polymeric network. Moreover, the maximum release efficiency in alkaline medium was between 92% and 98%, after 48 h, for both types of NPs. However, it can be noticed that the Cis loaded in the PCL-based NPs (NPs6) has smaller release efficiency than the Cis from the PNVP-based NPs (NPs4). This difference it might be attributed to the different nature of the two polymers; PNVP is hydrophilic and thus it swells in water leading thus to the release of Cis by a diffusion mechanism whereas the PCL is hydrophobic and the release of drug has been supposed to be a dilution controlled mechanism [40]. Furthermore, the Cis release behavior is in concordance with the results obtained for the swelling degree.

Having the data from the release kinetics of Cis from both types of NPs, it was of interest to obtain deeper insights about the drug transport mechanisms, therefore Table 2 summarizes the calculated parameters of kinetic Korsmeyer–Peppas equation describing the process of Cis release from both PNVP and PCL-based NPs.

As observed in Table 2, the release data of Cis from NPs4, which are the PNVP-based NPs, is fitted to the Korsmeyer–Peppas equation with a quasi-Fickian release exponent (*n* = 0.22), suggesting that the drug release from the NPs occurs primarily via diffusion. On the contrary, the release exponent n is equal cu 0.60 for the release data of Cis from NPs6 (PCL-based NPs) indicating an anomalous transport in which the release is controlled by both diffusion and erosion of the matrix.

### 3.4. In Vitro Evaluation of Nanoparticles Biocompatibility with the Blood Components

**Figure 3:**
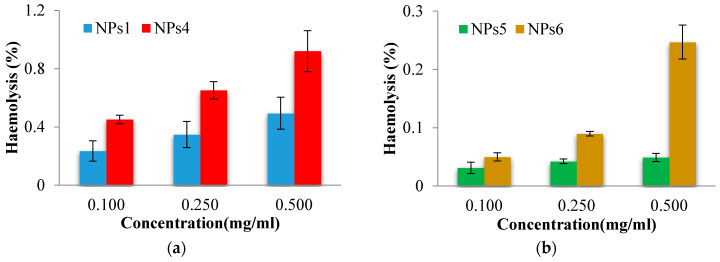
In vitro hemolysis assay for drug free and drug-loaded PNVP (**a**) and PCL-based (**b**) NPs.

Hemolysis is the destruction of red blood cells along with the release of hemoglobin and other internal components into the surrounding fluid. If this destruction occurs in a significant number of red blood cells in the body, it can lead to dangerous pathological conditions [41,42]. Therefore, all biomedical products designed for intravenous administration should be evaluated for their hemolytic potential [43]. As the obtained NPs were designed to be administered intravenously, preliminary tests in order to evaluate their interaction with human blood components were necessary. The obtained results concerning the hemolytic toxicity assay of NPs, after 3 h of incubation at different concentrations, are reported in Figure 3.

From the Figure 3, it clearly appears that the hemolysis was lower than 5%, for all tested NPs at all concentrations. A sample is considered as hemolytic if the hemolytic percentage is above 5% [44]. Moreover, as expected, it can be observed that the drug-loaded NPs show a higher hemolytic effect due to the presence of drug. Furthermore, the PCL-based NPs exhibit lower hemolytic effect than the NPs based on PNVP. Taking into account the obtained results it can be stated that the tested NPs are suitable for systemic administration.

### 3.5. Determination of the Cell Viability by the MTT Method

**Figure 4:**
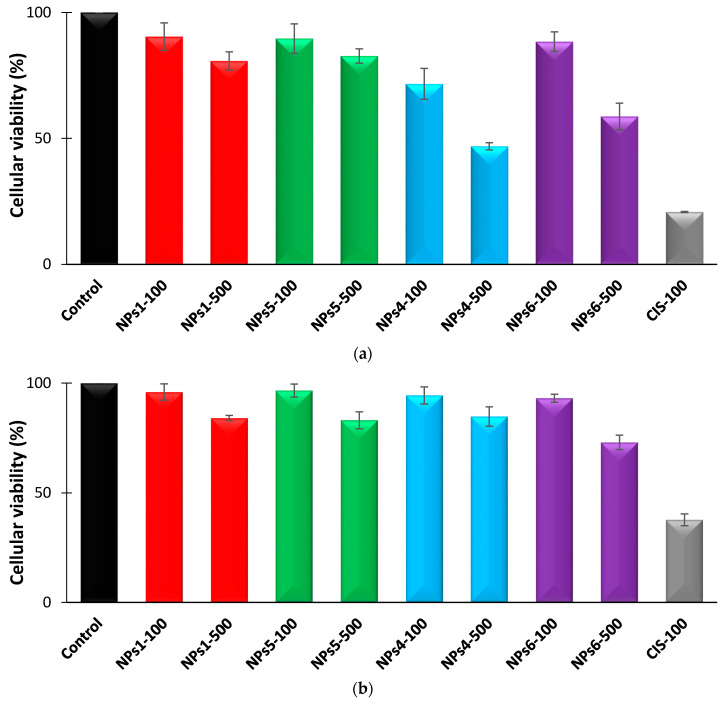
Evolution of the cellular viability after a treatment of 48 h with different NPs samples at two concentrations, 100 and 500 µg/mL. (**a**) A375 and (**b**) MCF7 tumor cells.

In Vitro studies, on A375 and MCF7 tumor cell cultures, aimed to investigate the biocompatibility of the non-loaded PNVP and PCL-based NPs (NPs1 and NPs5), which were subsequently loaded with Cis (NPs4 and NPs6), and to evaluate the efficiency of the cytostatic activity of Cis released from the NPs, using a treatment time of 48 h and doses ranging from 100 to 500 µg/mL. These results were subsequently compared to those obtained after the administration of free Cis. The evolution of the cellular viability at minimum and maximum concentration values (100 and 500 µg/mL), after a treatment of 48 h, for both tumor cell lines is given in Figure 4**.** The illustration of the same evolution after a treatment of 24 h is given in Figure S2 in Supplementary Materials.

From Figure 4, it appears that, in the case of A375 malignant melanoma cells, the treatment with the non-loaded NPs induced a moderate effect on the cell viability. Thus, the cell viability reaches, compared to the control, at the dose of 100 µg/mL the values of 90.41% for NPs1 and of 82.61% for NPs5, corresponding to values of cytotoxicity of 9.59% and respectively of 17.39%. The same tendency was observed at higher concentration, such as 500 µg/mL, where the cell viability values were equal to 80.74% for NPs1 and to 89.72% for NPs5, being correlated with values of 19.26% and 10.28% of cytotoxicity. From these results, it clearly appears that both types of non-loaded NPs are practically non-cytotoxic.

The treatment with drug-loaded NPs, samples NPs4 and NPs6, resulted in a significant decrease in cell viability, in a dose-dependent manner. At the minimum used dose (100 µg/mL) the cell viability values of 71.65% (NPs4) and 88.43% (NPs6) were recorded, corresponding to a cytotoxic effect of 28.35% and 11.57%, respectively. At the maximum administered dose (500 µg/mL), the cell viability values of 46.83%, for NPs4 sample, and 58.72%, for NPs6, were recorded, and correlated with cytotoxic effect values of 53.17% and 41.28%, respectively.

Concerning the treatment of A375 malignant melanoma cells for 48h with free Cis it was observed a strong impact on cell viability, being recorded values of 21.84% (at minimum dose of 10 µg/mL) and 20.76% (at maximum dose of 100 µg/mL), correlated with significant cytotoxic effects, of 78.16% and 79.24% respectively. At this point it has to be noted that a material is considered to be cytotoxic if the values of the inhibition of cellular processes are higher than 50%.

The same type of test was also performed on another tumor cell line, MCF7 (Figure 4b), derived from a breast adenocarcinoma. Treatment for 48 h with the same doses of non-loaded NPs1 and NPs5 resulted in insignificant cell viability decreases. Thus, it was found that in the case of the non-loaded NPs, the cell viability reached, at the maximum applied dose (of 500 µg/mL), values of 84.05% for NPs1 and of 83.03% for NPs5, correlated with cytotoxicity values of 15.95% and 16.97%, being well tolerated by the cells.

Unlike the A375 tumor cell line, the 48 h treatment of MCF7 cancer cells with Cis-loaded NPs4 and NPs6 was characterized by a lower interference with cell viability, being registered at the maximum dose the values of 84.76%, and of 72.97%, respectively, which corresponds to cytotoxic effects of 15.24% and 27.03%, respectively. The 48 h treatment with free Cis resulted in a marked decrease of the cell viability, reaching at maximum used dose value of 37.73%, correlated with a significant cytotoxic effect of 62.27%, which surpasses the threshold of minimum 50%, recommended by in vitro screening programs.

The experimental results confirm, for both tumor cell lines, the good cell compatibility of the both types of NPs, which are nontoxic and well tolerated by the cells. Comparing the two types of NPs loaded with Cis, it can be concluded that the NPs4, which is the PNVP-based NPs, shows faster release of the drug, which was achieved during the 48 h of treatment, but with lower antitumor efficacy than that of free Cis. For the same sample, a more pronounced cytotoxic effect was observed in the A375 tumor cell line. Finally, as expected, the cellular viability values are smaller after 48 h than those obtained for a treatment duration of 24 h.

### 3.6. Evaluation of Cell Apoptosis

**Figure 5:**
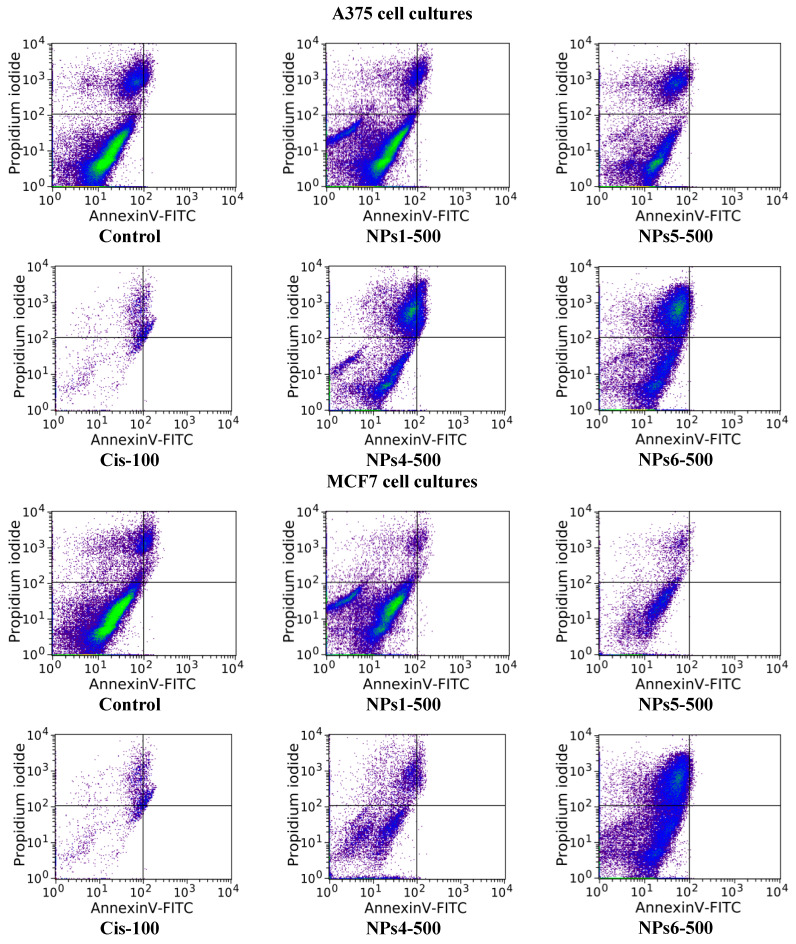
Effect of the 48 h treatment, at a concentration of 500 µg/mL of all the NP samples and Cis on the frequency distribution of viable (Q1), dead (Q2), apoptotic (Q3), and preapoptotic (Q4) cells corresponding to each group in the case of A375 and MCF7 neoplastic cell cultures.

**Figure 6:**
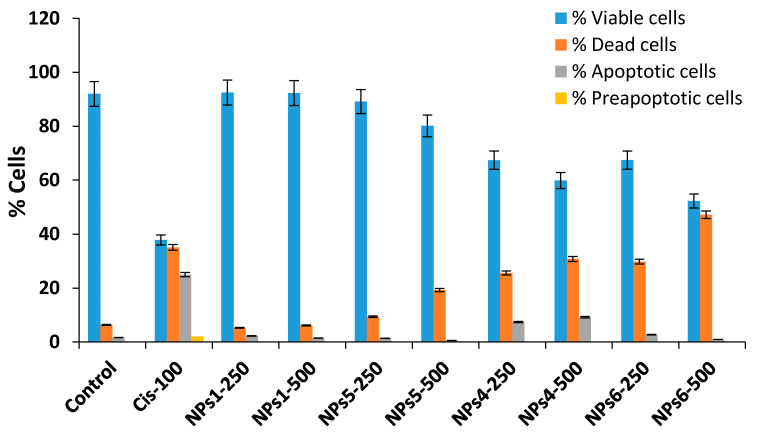
Comparison of the concentration effect of the 48-h treatment of all the NPs samples and Cis in the case of A375 neoplastic cell culture.

The cell apoptosis, for the both A375 and MCF7 tumor cell lines, was evaluated after a treatment of 48 h with the non-loaded NPs1 and NPs5 samples as well as with the Cis-loaded NPs4 and NPs6, at the doses of 250 and 500 µg/mL by Annexin V-FITC/propidium iodide assay [35,37]. The obtained results are provided in Figure 5 and Figure 6 as well as in Table S3 in Supporting Materials.

The impact of the treatment with the tested compounds, at different doses, on the frequency of different cell populations (living, preapoptotic, death, and apoptotic) is similar to that of the control in the case of samples without encapsulated drug NPs1 and NPs5 (at 250 µg/mL) (see Figure 5 and Figure 6). Moreover, the increasing of the concentration at 500 µg/mL has as consequence the reduction in cell viability which was evidenced by the increase in the number of dead cells. This increase was more important for sample NPs5 than for NPs1.

The drug-loaded samples, NPs 4 and NPs6, lead to significant increases in the frequency of dead and apoptotic cells compared to the control, indicating a significant impact on cell viability, these being dose dependent. It is worth noting that, at the maximum dose of the NPs4 sample, the percentage of apoptotic cells is similar for both tumor cell lines (9.24% for A375 and 9.60% in MCF7, respectively). This behavior can be justified by high sensitivity of the cells in S phase of the cell cycle. The cytotoxic mode of action of Cis is mediated by its interaction with DNA and leading up in the activation of apoptosis [38,45]. In the case of NPs6 sample, the apoptosis induction is smaller at a concentration of 500 µg/mL probably due to delayed release of the Cis from the drug delivery systems.

## 4. Conclusions

A series of drug-loaded non-aqueous dispersions, based on PCL and PNVP nanoparticles, were obtained starting from polymerizable non-aqueous monomer-in-silicone oil emulsions stabilized with the a tailor-made PDMS-based copolymer. These NPs, with sizes around 120 nm, were loaded with Cisplatin, an antitumoral model drug, directly in the polymerization step, and it was observed that the presence of the drug leads only to a slight increase of the NPs size. As expected, the swelling degree of the hydrophilic cross-linked PNVP-based NPs was directly correlated to the concentration of the cross-linking agent. The drug release kinetics were evaluated at 37 °C in phosphate buffer at pH = 7.4 and it appeared that the drug release rate from the hydrophilic cross-linked PNVP-based NPs is higher than that from the hydrophobic PCL-based NPs. Moreover, it was demonstrated that both types of NPs are hemocompatibles.

The in vitro experimental results, on A375 human malignant melanoma cell and MCF7 human breast carcinoma cells, have shown that the Cis-loaded PNVP and PCL-based NPs (NPs4 and NPs6) have exerted antitumor activity by increasing frequency of dead and pre- or apoptotic cells. Even if the apoptosis process is detected in a smaller proportion than the cytotoxic one, at the maximum concentration of Cis-loaded NPs, a significant impact has occurred. These results highlighted the significant cytotoxic impact of Cis-loaded NPs suggesting a possible biomedical applicability in controlled therapy of malignant tumor.

As a practical perspective of this study, the continuous silicone oil phase will be replaced with a functionalized PDMS oil which will be photopolymerized in order to obtain a biphasic biomaterial, as a thin film, with dispersed drug-loaded NPs in one batch. This biomaterial might be used as a patch or as a sub-cutaneous implant having a slow and controlled drug release.

## Figures and Tables

**Table 1:** Droplet and particle sizes as well as the PDI values for both non-aqueous emulsions and dispersions obtained at a stabilizer concentration of 3 wt % and a phase ratio of 10/90 VP or CL-in-PDMS

Sample	Dispersed Phase	Cross-Linking Agent (wt%) ^a^	DLS			
			Non-Aqueous Emulsion ^b^		Non-Aqueous Dispersion ^c^	
			Droplet Size (nm)	PDI	Particle Size (nm)	PDI
NPs1	VP	1	122.5 ± 0.9	0.03	126.3 ± 1.0	0.03
NPs2	VP	2	121.2 ± 2.0	0.01	128.3 ± 1.5	0.02
NPs3	VP	3	117.7 ± 2.0	0.02	132.6 ± 0.5	0.05
NPs4	VP+Cis	1	142.0 ± 1.8	0.04	148.0 ± 1.5	0.06
NPs5	CL	-	120.9 ± 3.0	0.04	132.1 ± 0.8	0.05
NPs6	CL+Cis	-	137.5 ± 1.8	0.06	148.8 ± 1.2	0.07

^a^ the cross-linking agent was the MBAA and the concentration is expressed with respect to the monomer, VP. ^b^ DLS values obtained, after dilution, for the VP and CL-in-PDMS oil non-aqueous emulsion. ^c^ DLS values obtained, after dilution, for the PNVP and PCL-based NPs dispersed in the PDMS oil.

**Table 2:** Basic kinetic parameters of the process of Cis kinetics release from both PNVP and PCL-based NPs

Sample	n	K	R^2^
NPs4	0.22	1.65	0.957
NPs6	0.60	2.54	0.972

## Supplementary Materials

The following are available online at https://www.mdpi.com/2073-4360/12/5/1018/s1. Figure S1: Evolution of the cellular viability after 24 h treatment with different NPs samples at two concentrations, 100 and 500 µg/mL. (a) A375 and (b) MCF7 tumor cells; Table S1: Effect of 48 h of treatment, with different concentrations (µg/mL) of the NPs1, NPs4, NPs5, and NPs6 samples, as well as of the cytostatic Cis on the frequency distribution of viable, dead, preapoptotic, and apoptotic cells corresponding to each batch, in the case of A375 and MCF7 neoplastic cell cultures.
