# Supplementary material for: Antitumoral Drug-Loaded Biocompatible Polymeric Nanoparticles Obtained by Non-Aqueous Emulsion Polymerization
Source: Polymers (Basel). 2020 Apr 30;12(5):1018. doi: 10.3390/polym12051018 (PMC7284542; doi:10.3390/polym12051018)
Supplement: Supplementary file 1 [file polymers-12-01018-s001.pdf]

Article

Antitumoral Drug-Loaded Biocompatible Polymeric Nanoparticles Obtained by Non-Aqueous Emulsion Polymerization

Oana Maria Daraba [1], Anca Niculina Cadinoiu [1], Delia Mihaela Rata [1], Leonard Ionut Atanase [1,*], and Gabriela Vochita [2]

[1] Department of Biomaterials, Faculty of Medical Dentistry, "Apollonia" University of Iasi, Pacurari Street, No. 11, Iasi 700511, Romania; maria.mary2019@yahoo.com (O.M.D.); jancaniculina@yahoo.com (A.N.C.); iureadeliamihaela@yahoo.com (D.M.R.)

[2] Department of Experimental and Applied Biology, NIRDBS—Institute of Biological Research Iasi, Lascar Catargi 47, Iasi 700107, Romania; gabriela.vochita@icbiasi.ro

* Correspondence: leonard.atanase@yahoo.com

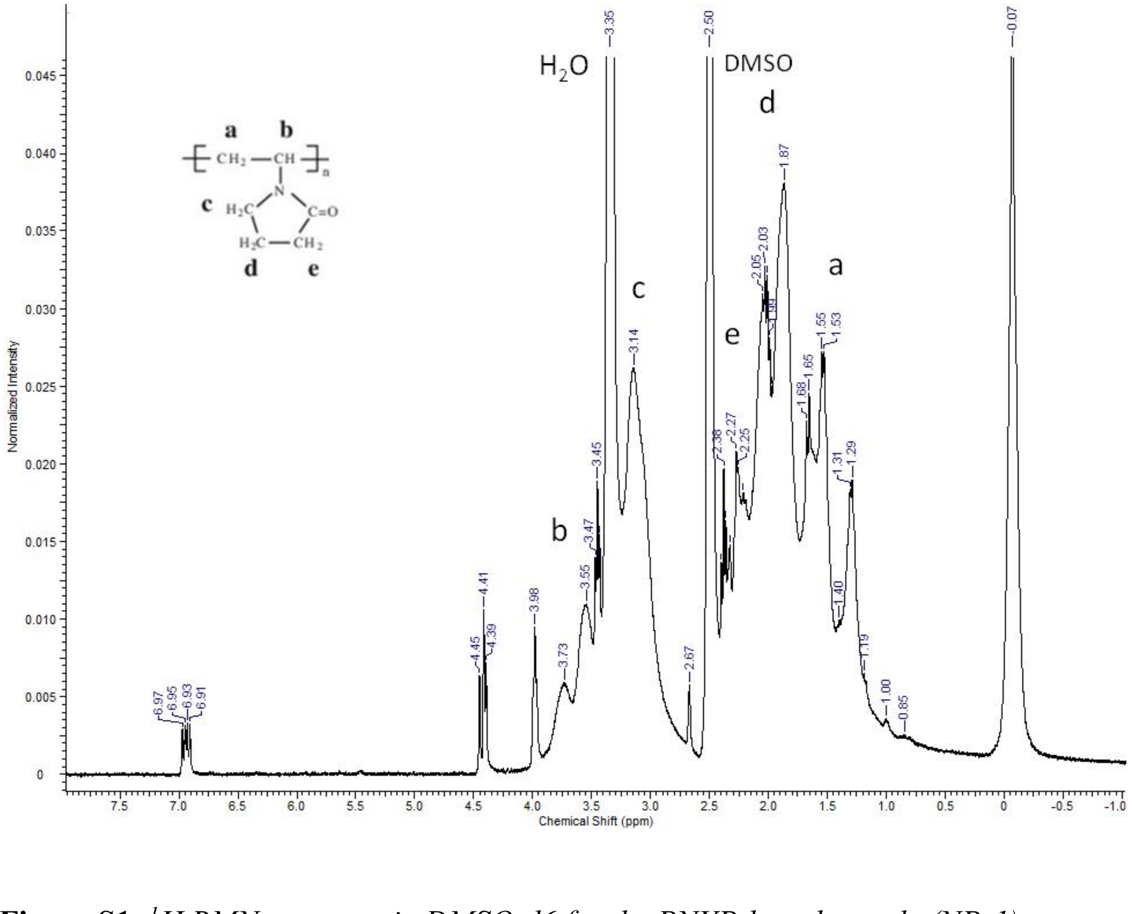

Figure S1: *^{1}H RMN spectrum in DMSO-d6 for the PNVP-based sample (NPs1)*

Table S1: *Peak assignments from the FT-IR spectrum of the PNVP-based NPs*

Wavelength (cm^{-1})	Groups
3433.15	O-H
2960.61	C-H asymmetrical
1651	C=O
1427.27	CH_2
1261.39	C-N
1087.81	Si-O-Si
796.57	Si-$(CH_3)_2$

Table S2: *Peak assignments from the FT-IR spectrum for the PCL-based NPs*

Wavelength (cm^{-1})	Groups
3433.15	O-H
2864-2944	C-H
1721	C=O
961-1471	CH_2

Table S4: *Molar mass and Tm of the PNVP and PCL-based NPs*

Sample	Dispersed phase	Cross-linking agent (wt%)[a]	Mn (SEC) (g/mol)[a]	Tm (˚C)
**NPs1**	VP	1	n.d	79
**NPs2**	VP	2	n.d	82
**NPs3**	VP	3	n.d	88
**NPs4**	VP+Cis	1	n.d	80
**NPs5**	CL	-	8600	63.7
**NPs6**	CL+Cis	-	5900	61.2

[a] PS equivalents

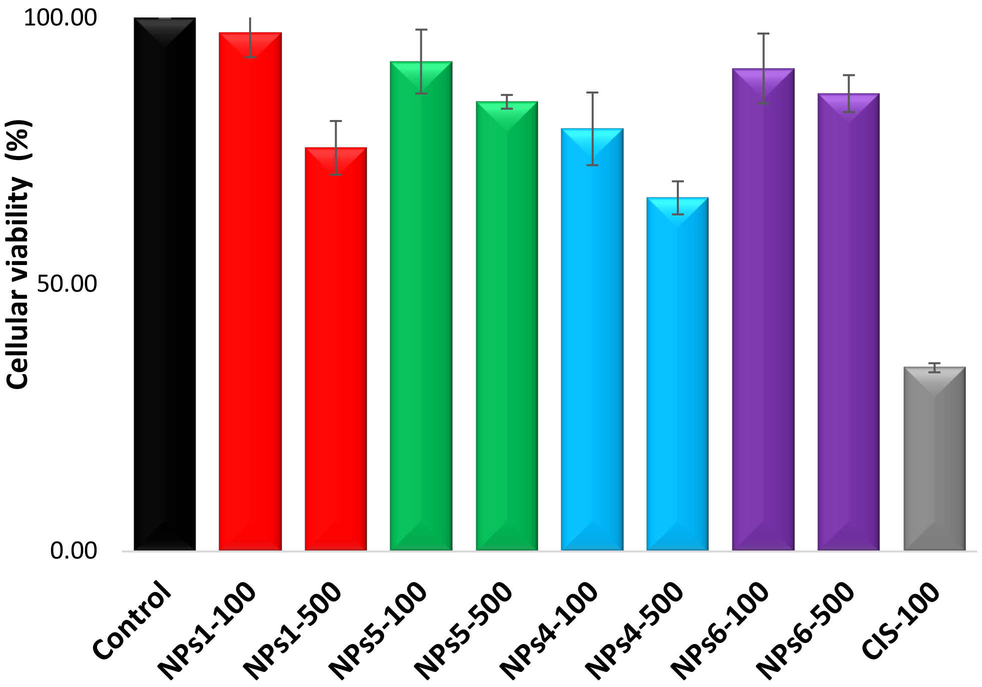

(a)

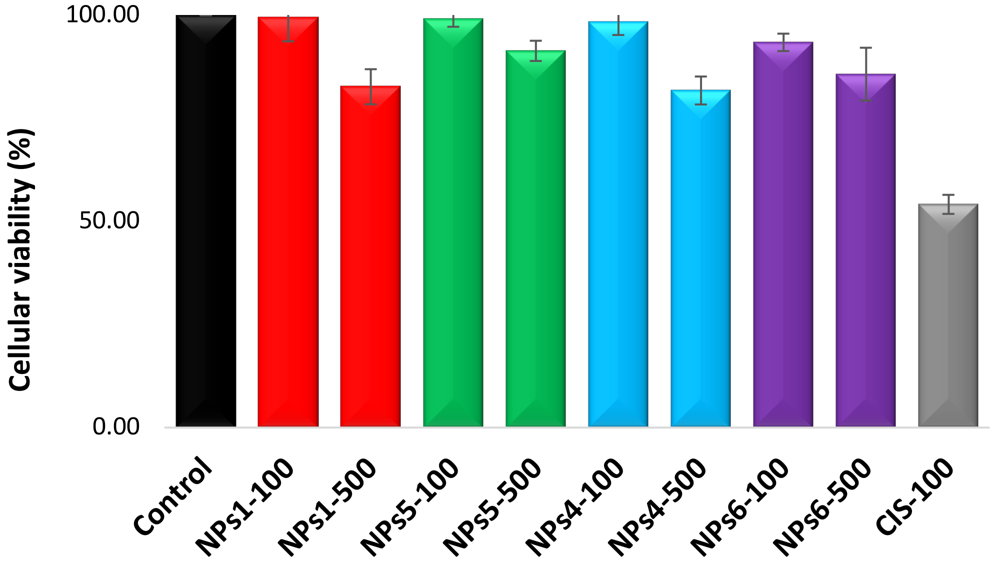

(b)

Figure S2: Evolution of the cellular viability after a treatment of 24h with different NPs samples at two concentrations, 100 and 500 µg/mL. (a) A375 and (b) MCF7 tumor cells

Table S4: The effect of 48h of treatment, with different concentrations (μg/ml) of the NPs1, NPs4, NPs5 and NPs6 samples, as well as of the cytostatic Cis on the frequency distribution of viable, dead, preapoptotic and apoptotic cells corresponding to each batch, in the case of A375 and MCF7 neoplastic cell cultures.

**A375**	**Control**	**NPs1-250**	**NPs1-500**	**NPs5-250**	**NPs5-500**	**NPs4-250**	**NPs4-500**	**NPs6-250**	**NPs6-500**	**CIS 100**
% Viable cells	91.99	92.45	92.31	89.16	80.11	67.39	59.86	67.43	52.32	37.84
% Dead cells	6.33	5.25	6.16	9.40	19.28	25.62	30.79	29.81	47.19	35.09
% Apoptotic cells	1.66	2.25	1.45	1.42	0.60	7.42	9.24	2.72	0.96	25.01
% Preapoptotic cells	0.02	0.06	0.09	0.03	0.02	0.08	0.12	0.04	0.02	2.07
**MCF7**	**Control**	**NPs1-250**	**NPs1-500**	**NPs5-250**	**NPs5-500**	**NPs4-250**	**NPs4-500**	**NPs6-250**	**NPs6-500**	**CIS 100**
% Viable cells	95.67	96.15	96.00	91.83	83.31	70.08	62.25	70.12	54.41	39.35
% Dead cells	3.52	2.92	3.42	7.55	16.00	26.23	28.06	28.00	40.56	38.98
% Apoptotic cells	0.92	1.25	0.80	0.79	0.66	3.98	9.56	1.84	3.77	19.49
% Preapoptotic cells	0.02	0.06	0.09	0.03	0.02	0.08	0.13	0.04	1.26	2.17
